# *TP53* expression in PCAT in coronary artery disease combined with type 2 diabetes mellitus and its correlation with CCTA radiomic features: novel imaging biomarkers

**DOI:** 10.3389/fmed.2025.1626390

**Published:** 2025-09-22

**Authors:** Haicheng Qi, Yahui Hu, Yan Li, Xiumei Li, Yan Xing

**Affiliations:** ^1^Imaging Center, The First Affiliated Hospital of Xinjiang Medical University, Urumqi, China; ^2^Department of Pain Medicine, Midong District Hospital of Traditional Chinese Medicine, Urumqi, China; ^3^School of Basic Medical Science, Xinjiang Medical University, Urumqi, China

**Keywords:** coronary artery disease, type 2 diabetes mellitus, pericoronary adipose tissue, radiomics, *TP53*

## Abstract

**Aims:**

To explore the changes in differentially expressed genes in pericoronary adipose tissue (PCAT) and serum from patients with coronary artery disease (CAD) complicated with type 2 diabetes mellitus (T2DM) and to analyse its correlation with PCAT radiomic features based on coronary CT angiography (CCTA).

**Methods:**

Intersecting genes that were differentially expressed in both CAD and T2DM patients were obtained from the GEO database and analyzed to obtain candidate genes. PCAT and serum samples were collected from CAD patients who underwent coronary artery bypass grafting (CABG) from May 2023 to January 2024. RT–qPCR was used to determine the expression of candidate differentially expressed genes in PCAT, to search for genes related to patients with CAD combined with T2DM, and to verify the protein expression levels by immunohistochemistry (IHC). Enzyme-linked immunosorbent assays (ELISAs) were also used to determine the expression of candidate differentially expressed genes in the serum. Finally, the PCAT radiomic features of the right coronary artery in patients with CAD combined with T2DM were extracted and correlated with the candidate genes.

**Results:**

*HLA-DRB1*, *TP53*, and *CCR9* were screened from the GEO database. RT–qPCR results revealed that *TP53* expression was significantly increased in the T2DM group compared with the control group (3.082 ± 0.580 vs. 1.663 ± 0.698, *p* < 0.001). IHC results revealed that the area of positive expression around the nucleus was increased in the fat cells of the T2DM group compared with those of the control group, with increased perinuclear areas with positive expression (0.521 ± 0.082 vs. 0.327 ± 0.074, *p* < 0.001), and 14 PCAT radiomic features in CAD combined with T2DM patients correlated with *TP53* (r_s_ > 0.5, *p* < 0.05).

**Conclusion:**

*TP53* expression was significantly elevated in the PCAT of patients with CAD combined with T2DM, suggesting that this molecule plays a role in the development of this disease. Four first-order features and 10 texture features in the PCAT radiomic features were associated with abnormal *TP53* expression. The association of radiomic features with *TP53* suggests that CCTA-based radiomic features can be used to predict abnormalities in differential gene expression, thus providing a new way to noninvasively predict CAD combined with T2DM.

## 1 Introduction

According to the World Health Organisation, 17.9 million people worldwide suffer from cardiovascular diseases. Coronary artery disease (CAD) is the third most prevalent cardiovascular disease and is increasing annually; thus, CAD is a serious threat to human life and health (1, 2). Notably, a close pathophysiological link between CAD and diabetes mellitus has been found, and the two share multiple risk factors, including obesity, insulin resistance, and chronic inflammation, forming a mutually reinforcing vicious cycle (3). Type 2 diabetes mellitus (T2DM) accounts for 96% of all diabetes mellitus cases (4). The *TP53* gene plays a crucial role in diseases such as T2DM and CAD, primarily by inducing inflammatory responses that influence the progression of atherosclerosis (5). Therefore, elucidating the role of *TP53* in these diseases may offer new insights into the pathophysiological mechanisms underlying CAD combined with T2DM. The comorbid state of CAD and T2DM substantially exacerbates the clinical risk, and evidence-based medicine has demonstrated that patients with CAD in combination with T2DM are at increased risk for adverse cardiovascular events and have a worse prognosis and a greater risk of death; CAD is also the leading cause of death and disability (6). Therefore, early and precise treatment is particularly important for patients with CAD combined with T2DM.

Currently, coronary computed tomography angiogram (CCTA) has been used as a first-line imaging method for CAD (7, 8), and CCTA-detected attenuation of coronary arteries in pericoronary adipose tissue (PCAT) has been shown to reflect vascular inflammation, which is a key factor in the formation, progression and rupture of coronary atherosclerotic plaques (9, 10). Long-term glycaemic and metabolic abnormalities in patients with T2DM lead to a long-term inflammatory state in coronary blood vessels, and the release of free fatty acids from PCAT increases, which further damages endothelial cells and accelerates coronary atherosclerosis (11). The PCAT attenuation index was found to be significantly greater in diabetic patients than in nondiabetic patients (12). Therefore, this method can be used to predict the occurrence of CAD combined with T2DM by detecting changes in PCAT via CCTA. CCTA-based PCAT radiomic features are able to mine quantitative features from images, reducing the need for invasive diagnostics (13). Oikonomou et al. (14) investigated the associations of CCTA-based PCAT radiomic features with genes related to a high risk of perivascular structural remodelling in CAD patients, confirming the adverse cardiovascular event risk of fat radiomic features. Therefore, correlation analysis between radiomic features and genes has potential for predicting CAD in combination with T2DM and may provide new ideas for clinical decision-making.

In this study, PCAT was used as an entry point and combined with gene bioinformatic analysis to screen key genes and perform basic experimental validation. Moreover, a correlation analysis of gene expression with PCAT radiomic features was performed to identify new imaging biomarkers for the risk prediction of CAD combined with T2DM.

## 2 Materials and methods

### 2.1 Study subjects

Pre-coronary artery bypass grafting (CABG) CAD patients who underwent CCTA from May 2023 to January 2024 were prospectively and consecutively enrolled at the First Affiliated Hospital of Xinjiang Medical University and grouped according to whether they had T2DM. The study was approved by the Medical Ethics Committee of the hospital (Ethics Approval No. 220120-02), and the patients and their families signed an informed consent form. The inclusion criteria were as follows: (1) age >18 years; (2) definite clinical diagnosis of CAD; (3) CABG within 1 month after CCTA examination; and (4) T2DM, defined as a known history of previous diabetes, current use of medication for diabetes (oral or injectable medication), and a glycated haemoglobin ratio (HbA1c) ≥ 6% for at least 1 month prior to study registration. The exclusion criteria were as follows: (1) the samples were not frozen in a liquid nitrogen tank for more than 10 min; (2) the RNA concentration was too low; (3) the samples were from people with serious illnesses such as tumours, blood and immune system diseases; (4) the samples had artefacts in the image that could not be used for diagnosis; (5) the samples were from patients with an allergy to an iodine contrast agent; (6) the samples were from patients with severe renal insufficiency; and (7) the samples were from patients with incomplete clinical data.

**Figure 1:**
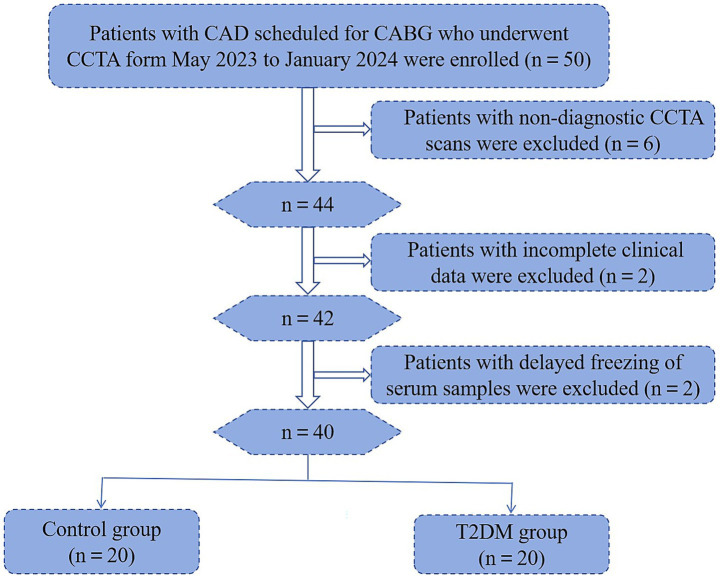
Flowchart of participant enrolment.

**Figure 2:**
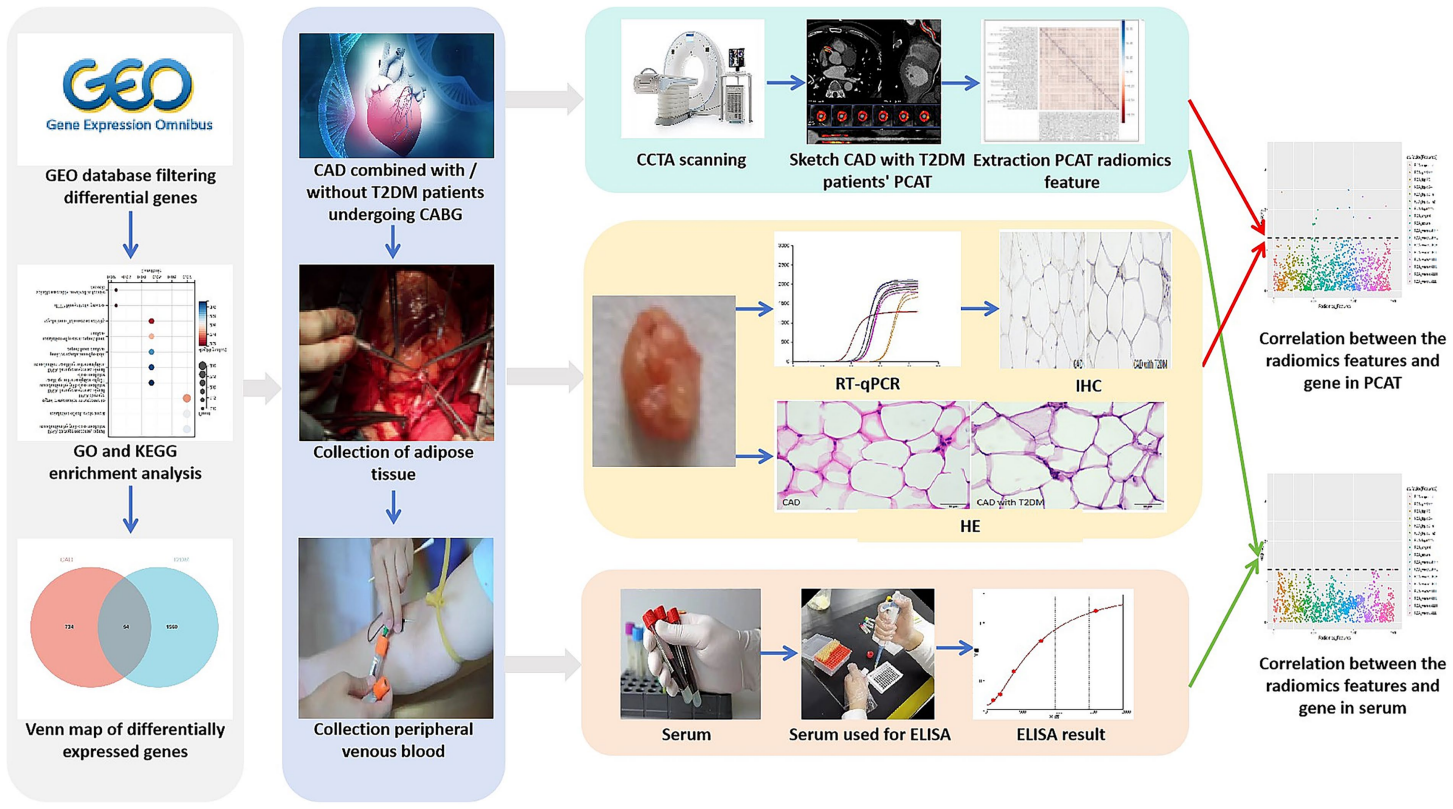
Research flow chart.

In this study, six patients whose CCTA scans failed, two patients whose clinical data were incomplete, and two patients whose serum samples were not frozen in time were excluded; finally, PCAT, peripheral blood and clinical data were collected from 40 patients (Figure 1). The flow chart of the study is shown in Figure 2.

### 2.2 CCTA scanning scheme

CCTA examinations were performed using a 320-row detector CT (Aquilion ONE Genesis Edition, Canon Medical Systems, Otawara, Japan) with volumetric scanning, prospective acquisition of multiphase images with 30%–80% R–R intervals, and scanning ranging from 1 cm below the tracheal eminence to 1.5 cm below the cardiac diaphragm. The scanning area ranged from 1 cm below the tracheal eminence to 1.5 cm below the cardiac diaphragm. The tube voltage was 120 kV, the tube current value was controlled by automatic exposure, the rotational speed of the bulb tube was 0.275 *r*/s, the thickness of the layer was 0.5 mm, the spacing of the layers was 0.25 mm, and the reconstruction matrix was 512 × 512. The contrast injection scheme was based on the regiment of interest (ROI) method: the ROI in the descending aorta at the level of the centre of the heart was set as the threshold monitoring point. When the descending aorta contrast concentration reached 280 HU, the scan was automatically triggered with a 1 s delay. During the enhancement scan, a nonionic contrast agent (iohexol or iopamidol injection with an iodine concentration of 370 mg/mL) was injected intravenously through the elbow vein at a rate of 3.5–5.0 mL/s; 30 mL of saline was injected at the same rate. Dose of iodine contrast (mL) = body weight (kg) × 0.8 mL/kg (15).

### 2.3 Screening of candidate genes in CAD-integrated T2DM

CAD datasets (GSE64554 and GSE120774) and T2DM datasets (GSE16415 and GSE71416) were first downloaded from the GEO database,^1^ and Sangerbox^2^ to merge, decatch and normalise the datasets, the limma analysis tool for differentially expressed gene analysis, and the Sangerbox Wayne plotting tool to obtain intersecting genes that are differentially expressed in both CAD and T2DM were used. The DAVID Functional Annotation Bioinformatics Microarray Analysis website^3^ was used for Gene Ontology (GO) functional annotation with Kyoto Encyclopaedia of Genes and Genomes (KEGG) analysis to obtain candidate genes.

### 2.4 RT–qPCR analysis of candidate gene expression in PCAT

The genes most relevant for CAD combined with T2DM were identified via basic experimental validation. First, RT–qPCR experiments were performed on PCAT samples from the T2DM group and the control group. The collected adipose tissue samples were stored at −80 °C, RNA was extracted via TRIzol (Thermo Fisher), RNA was transcribed into cDNA via a cDNA reverse transcription kit (TaKaRa), and the concentration of RNA was determined by a Thermo Fisher Nanodrop 2000 at 260/280. Gene expression was determined via the Quant Studio TM 6 real-time PCR system. *β*-actin was used as a reference gene, and relative mRNA expression was determined by the 2^-ΔΔCt^ method. The primers used are shown in Table 1.

**Table 1:** Primer sequences.

Primer name	Primer sequence (5′ → 3′)
*GAPDH*	F: ACAGCCTCAAGATCATCAGCAA R: CCATCACGCCACAGTTTCCC
*HLA-DRB1*	F: ACTGTGTATCCTTCAAAGACCCA R: CAATGCTGCCTGGATAGAAACC
*TP53*	F: ACATTCTCCACTTCTTGTTCCCC R: CCCCACAACAAAACACCAGT
*CCR9*	F: AAGAGTGAAGACCATGACCG R: TTTCTCCCTCCAAGTATGTGC

### 2.5 Immunohistochemistry (IHC) validation of the expression of candidate genes in PCAT

Genes screened by RT–qPCR were validated at the protein level (IHC). Frozen sections (10 mm) were air-dried for 15 min and then immersed in xylene for 10 min. Subsequently, the sections were rehydrated using gradient alcohol and stained with haematoxylin and eosin. IHC was performed on selected serial sections using the Universal Elite ABC Kit (Vector Laboratories) following the manufacturer’s guidelines. The sections were treated with a methanol solution of 0.3% H_2_O_2_ for 30 min and then blocked with 5% horse or goat serum. After PBS rinses, the sections were incubated with primary antibody in a humidity-controlled chamber for 1 h. The slides were incubated with secondary antibody for 30 min and then with anti-biotin protein-biotin for another 30 min. Finally, the sections were exposed to DAB and restained with haematoxylin. The antibody used in the experimental procedure was *TP53* (AF0879; Jiangsu Pro-Tech Biological Research Centre, Inc.; dilution ratio: 1:100; China).

### 2.6 Enzyme-linked immunosorbent assay (ELISA) for the validation of candidate genes in serum

Validation of CAD-incorporated T2DM-associated candidate genes at the serum level was conducted. Peripheral venous blood samples (10 mL) were collected in pyrogen-free tubes (with EDTA as an anticoagulant) prior to the start of somatic circulation. For serum preparation, EDTA tubes were placed on ice and centrifuged at 1500 × g for 10 min at 4 °C within 20 min. The resulting sera were aliquoted and stored at −80 °C for subsequent experimental assays. The expression levels of factors (*TP53*, *HLA-DRB1* and *CCR9*) associated with CAD combined with T2DM were determined using ELISA kits (Shanghai Tongwei Technology Co., Ltd., China).

### 2.7 PCAT radiomic feature extraction

**Figure 3:**
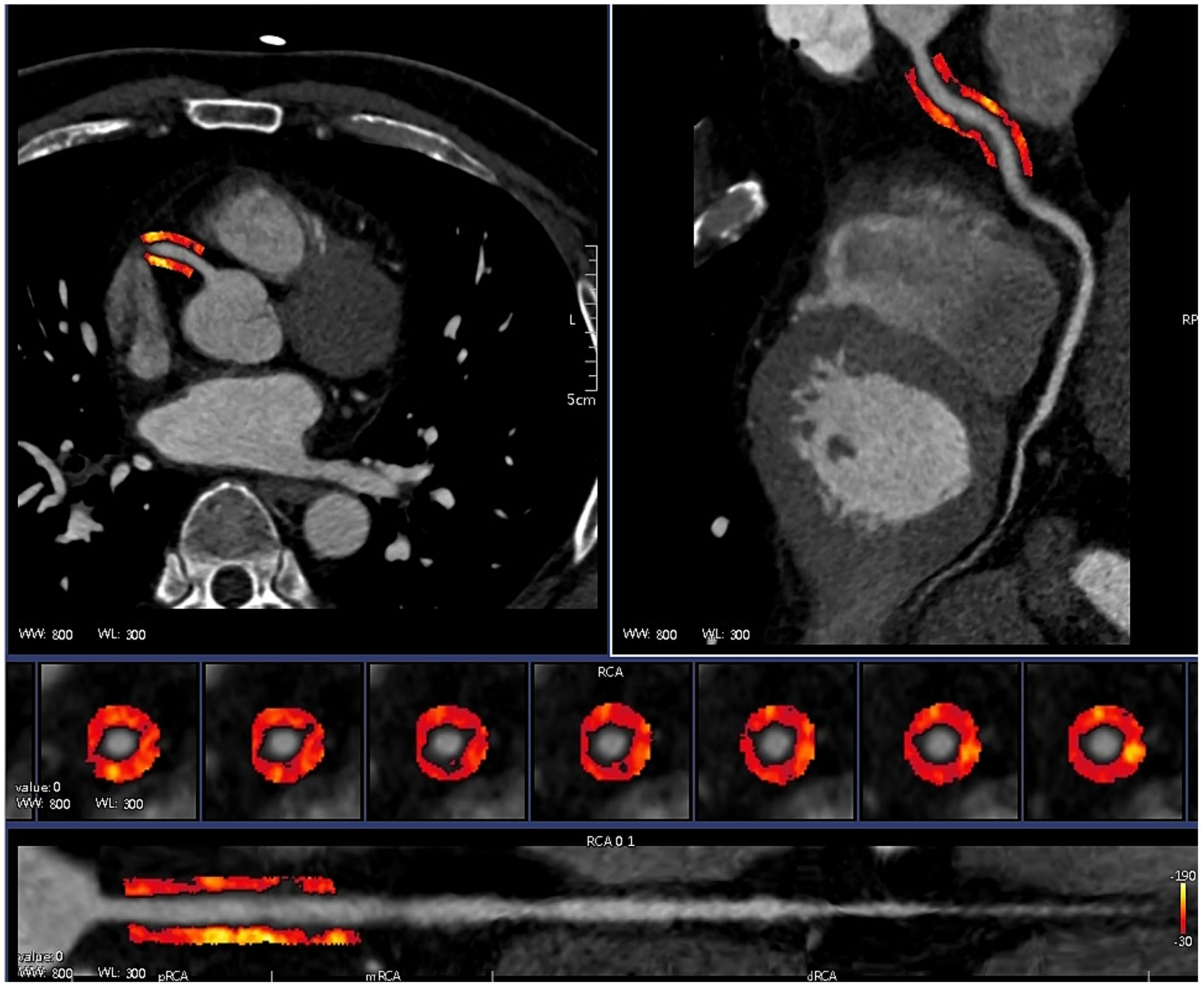
Automatic drawing of PCAT.

CCTA images of patients with CAD combined with T2DM were subjected to ROI segmentation of PCAT by selecting a 40 mm long and 3 mm wide section from 10 to 50 mm below the opening of the right coronary artery with a 1 mm gap around the coronary artery wall, using a Hounsfield unit threshold of −190 HU to −30 HU and using the centreline of the vessel as a baseline to ensure accurate segmentation of the PCAT (16) (Figure 3). The Perivascular Fat Analysis Tool software (UltraScholar’, Shukun Technology Co., Ltd., Beijing, China) was used to extract the PCAT radiomic features of the right coronary artery, which included first-order features, texture features, and shape features, and then, the original image was processed with seven filters, including the exponential, gradient, local binary (2D and 3D), logarithmic square and wavelet modes. This analysis integrates a comprehensive set of features. Features with the same radiomic feature values are eliminated by feature preprocessing, and the remaining features are subjected to feature preselection. Features with absolute values of Pearson correlation coefficients ≥ 0.9 were screened, and the remaining features were retained for analysis of CAD combined with PCAT radiomic features of T2DM patients for correlation analysis with genes.

### 2.8 Statistical analysis

Statistical analyses were performed using SPSS Statistics 26.0, and normality was first verified using the Shapiro–Wilk test for small sample sizes, with measurements conforming to a normal distribution expressed as the mean ± standard deviation ($\bar{x}\pm$s) and those not conforming to a normal distribution as [M(Q1, Q3)]. Counts are expressed as the number of cases and percentage. Two independent samples were tested using the independent samples *t-*test and Mann–Whitney *U* test on the basis of the normality of the results. Categorical variables were compared between groups using the chi-square test (*χ*^2^). *p* < 0.05 indicated a statistically significant difference. The strength of the association between gene expression and radiomic features conformed to a normal distribution according to Pearson’s analysis and did not conform to normality according to Spearman’s analysis. Manhattan plots were drawn using correlation *p* values [−log10(*p* values)] via the R language (R4.1.2; http://www.R-project.org).

## 3 Results

### 3.1 Patient characteristics

The clinical characteristics and medication data of all the study subjects are shown in Table 2. The control group and the T2DM group were predominantly male, and the CK values of the patients with CAD combined with T2DM were significantly greater than those of the patients with CAD (*p* = 0.040); otherwise, no significant differences were found between the general clinical data of the two groups.

**Table 2:** Clinical data of the control group and T2DM group patients.

Items	Control group (*n* = 20)	T2DM group (*n* = 20)	*p*
Male (%)	18 (90.00%)	17 (85.00%)	0.633
Age (years)	60.00 ± 5.99	57.80 ± 7.43	0.309
BMI (kg/m^2^)	25.70 ± 4.04	25.20 ± 3.59	0.712
TG (mmol/L)	1.31 (0.92,1.53)	1.63 (1.29,2.03)	0.076
TC (mmol/L)	3.41 (3.11,4.30)	3.18 (2.67,4.01)	0.277
HDL (mmol/L)	0.76 (0.69,1.01)	0.76 (0.60,0.90)	0.265
LDL (mmol/L)	1.85 (1.36,2.45)	1.76 (1.38,2.23)	0.659
ALT (U/L)	29.96 (18.00,32.75)	22.00 (18.10,27.22)	0.174
AST (U/L)	27.79 (22.93,34.12)	23.10 (20.61,27.62)	0.121
LDH (U/L)	146.16 (142.06,165.60)	146.11 (138.67,175.14)	0.738
CK (U/L)	50.89 (32.62,59.95)	70.39 (49.70,81.16)	0.040*
Risk factor (%)			
Hypertension	8 (40.00%)	11 (55.00%)	0.342
Smoking history	8 (40.00%)	10 (50.00%)	0.525
Drinking history	6 (30.00%)	3 (15.00%)	0.256
Cerebrovascular history	0 (0.00%)	3 (15.00%)	0.072
Family history	3 (15.00%)	1 (5.00%)	0.292
Drug use (%)			
Statins	14 (70.00%)	11 (55.00%)	0.327
Aspirin	13 (65.00%)	15 (75.00%)	0.490
Clopidogrel hydrochloride	6 (30.00%)	9 (45.00%)	0.327

TG, triglyceride; TC, total cholesterol; HDLc, high-density lipoprotein cholesterol; LDLc, low-density lipoprotein cholesterol; ALT, alanine aminotransferase; AST, aspartate transaminase; LDH, lactate dehydrogenase; CK, creatine kinase; *p < 0.05.

### 3.2 Acquisition of candidate genes associated with the occurrence of CAD combined with T2DM

**Figure 4:**
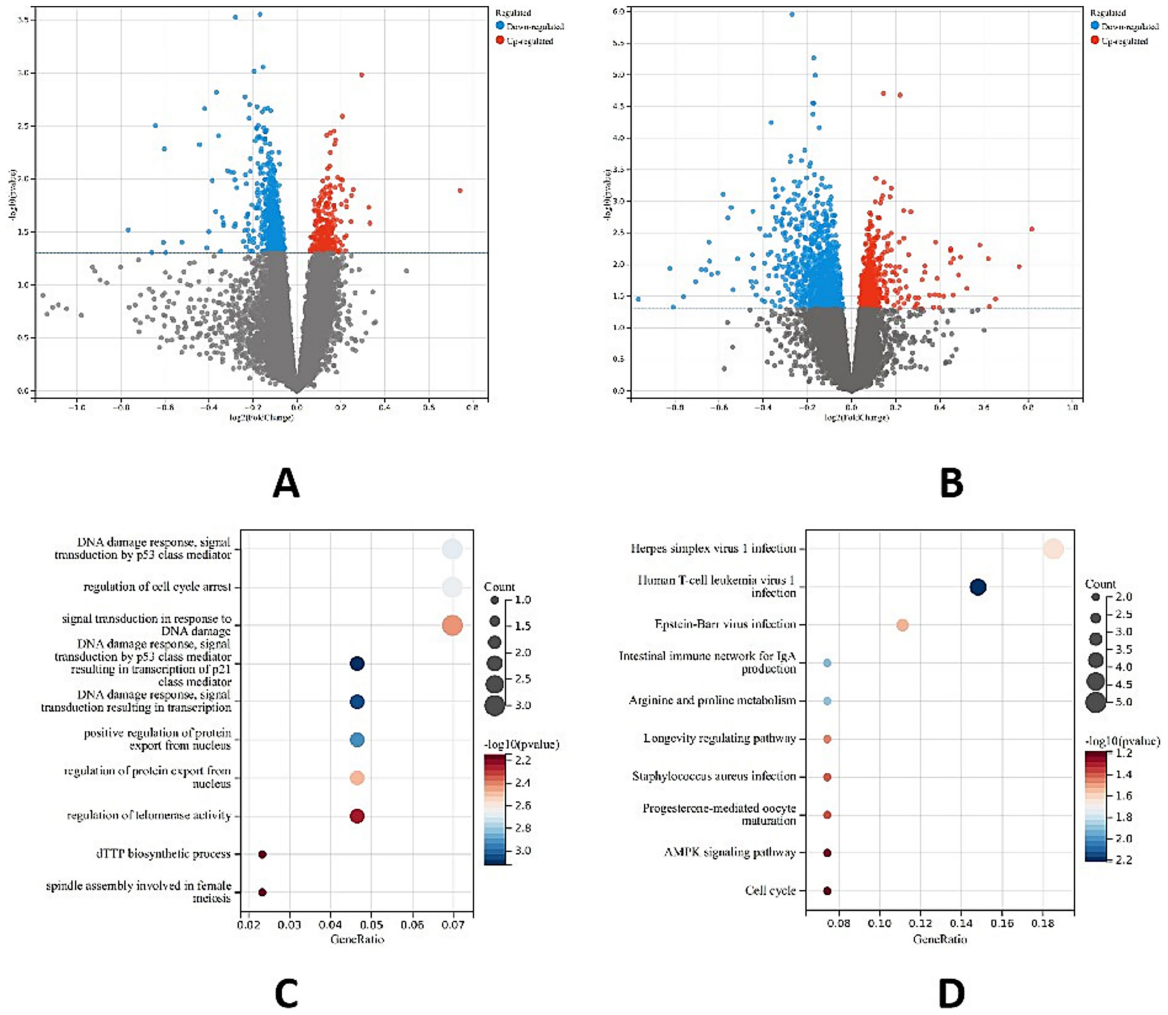
Differentially expressed genes in T2DM and CAD patients. **(A)** Volcano plot of genes differentially expressed between CAD patients and non-CAD patients; **(B)** volcano plot of genes differentially expressed between T2DM patients and non-T2DM patients; **(C)** GO functional enrichment analysis; **(D)** KEGG enrichment analysis.

In the CAD datasets GSE64554 and GSE120774, the differentially expressed genes were taken as intersections, and the number of common genes was 788. In the T2DM datasets GSE16415 and GSE71416, 1,614 common genes were identified. The differentially expressed genes of CAD and T2DM were intersected, and GO enrichment analysis was subsequently performed to filter out the eligible GO terms. A total of 180 KEGG pathways were enriched. The intersection of the two groups of differentially expressed genes was taken, and 54 common genes were obtained. Combined with GO functional annotation to select the differentially expressed genes in the inflammation and glycolipid metabolism categories, the genes obtained from the KEGG analysis were intersected, and three candidate genes related to the occurrence of CAD combined with T2DM were obtained: *HLA-DRB1*, *TP53*, and *CCR9* (Figure 4).

### 3.3 Gene-level validation of candidate genes in PCAT

**Figure 5:**
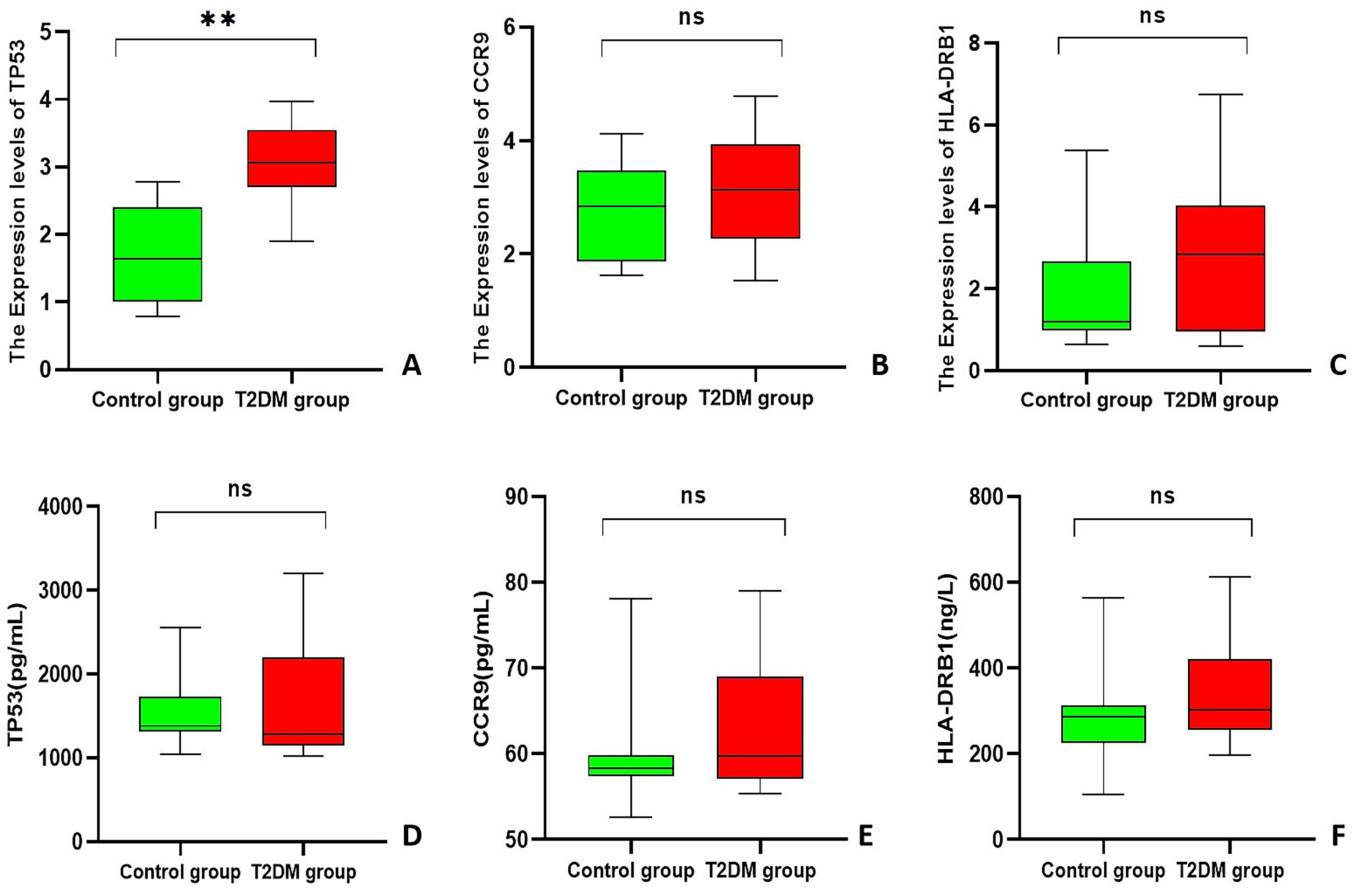
Box plot. **(A–C)** Statistical chart of the relative expression of genes in PCAT; **(D–F)** ELISA results for each gene. **Indicates *p* < 0.001.

A comparison of the T2DM group with the control group revealed that the relative expression of *TP53* was significantly upregulated in the T2DM group (3.082 ± 0.580) compared with that in the control group (1.663 ± 0.698) (3.082 ± 0.580 vs. 1.663 ± 0.698, *p* < 0.001); the relative expression of *HLA-DRB1* in the T2DM group (2.721 ± 1.824) was compared with that in the control group (1.804 ± 1.273), and these values were not significantly different between the two groups (2.721 ± 1.824 vs. 1.804 ± 1.273, *p* = 0.073); and the relative expression of *CCR9* in the T2DM group (3.031 ± 0.970) was compared with that in the control group (2.742 ± 0.851), and these values were not significantly different between the two groups (3.031 ± 0.970 vs. 2.742 ± 0.851, *p* = 0.324) (Figures 5A–C).

### 3.4 Validation of *TP53* at the protein level

**Figure 6:**
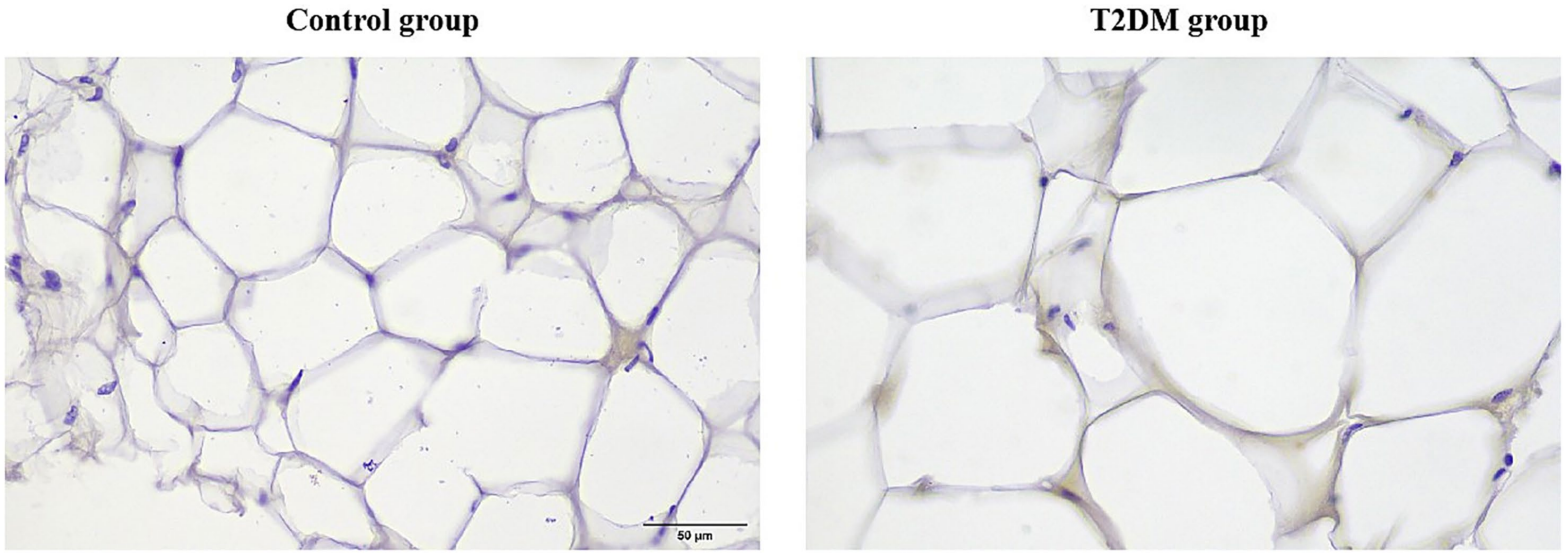
IHC results of *TP53* in PCAT (magnification: 40×).

On the basis of the RT–qPCR results, IHC was used to further verify the expression of *TP53* in PCAT. ImageJ analysis of the percentage of positively stained areas revealed that the area of *TP53* around the nucleus of adipocytes in the T2DM group was 0.521 ± 0.035 and that of the control group was 0.327 ± 0.074. The positive area of *TP53* around the nucleus of adipocytes in the T2DM group was significantly greater than that in the control group (0.521 ± 0.035 vs. 0.327 ± 0.074, *p* < 0.001) (Figure 6).

### 3.5 Validation of candidate genes at the serum level by ELISAs

The results of the ELISA experiments revealed that the expression of *TP53* was 1285.11 (1143.21, 2201.46) pg/mL in the T2DM group and 1381.43 (1315.19, 1730.82) pg/mL in the control group, and no statistically significant difference in the expression of *TP53* in the serum of the two groups of patients (*U* = 172, *p* = 0.449) was found. *CCR9* expression was 59.76 (57.06, 69.02) pg/mL in the T2DM group and 58.32 (57.38, 59.85) pg/mL in the control group, and no statistically significant difference in the expression of *CCR9* in the serum of the two groups of patients (*U* = 228, *p* = 0.449) was found. *HLA-DRB1* expression was 302.32 (256.24, 421.06) ng/L in the T2DM group and 287.11 (224.61, 312.69) ng/L in the control group, and no statistically significant difference in the expression of *HLA-DRB1* in the sera of the two groups of patients (*U* = 262, *p* = 0.094) was found (Figures 5D–F).

### 3.6 Morphological changes in PCAT adipocytes

**Figure 7:**
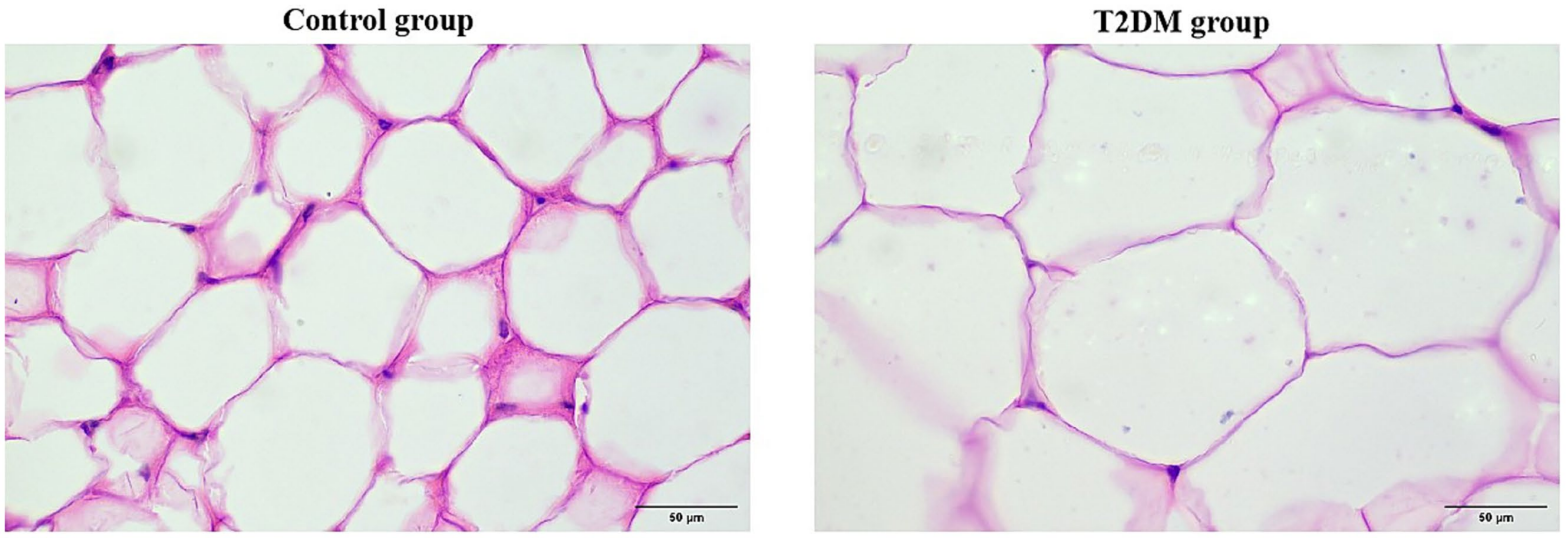
HE results of genes in PCAT (magnification: 40×).

HE staining of PCAT in patients with CAD combined with T2DM and ImageJ software analysis revealed that the unit cell area of PCAT adipocytes in the T2DM group was 6954.98 ± 1716.99 μm2, the unit cell area of PCAT adipocytes in the control group was 3278.15 ± 488.31 μm2, and this value in the T2DM group was significantly larger (6954.98 ± 1716.99 μm2 vs. 3278.15 ± 488.31 μm2, *p* < 0.001) than that in the control group (Figure 7).

### 3.7 PCAT radiomic features and *TP53* correlation

**Figure 8:**
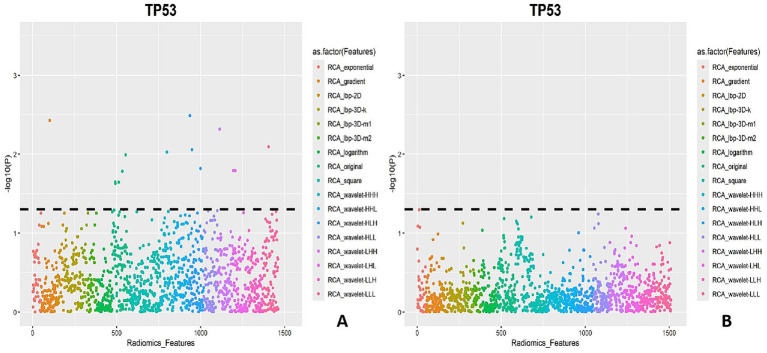
Manhattan plot. **(A)** Manhattan plot of the correlations between PCAT radiomic features and relative *TP53* expression; **(B)** Manhattan plot of the correlations between PCAT radiomic features and ELISA results.

A total of 1,688 PCAT radiomic features (first-order features, shape features, and texture features) were extracted from CAD-merged T2DM. There were 14 PCAT radiomic features that correlated with *TP53* (*r_s_* > 0.5, *p* < 0.05): four first-order features, 10 texture features, and no morphological features. The first-order features were wavelet-HLH_firstorder_Mean, wavelet-LHH_firstorder_Skewness, wavelet-LHL_firstorder_Energy, and wavelet-LHL_firstorder_TotalEnergy, and the texture features were gradient_gldm_LargeDependenceHighGrayLevelEmphasis, original_glcm_ClusterProminence, original_glcm_ ClusterTendency, original_gldm_DependenceEntropy, original_glrlm_RunEntropy, original_glszm_ZoneEntropy, wavelet-HHH_gldm_ DependenceVariance, wavelet-HLH_glcm_ClusterShade, wavelet-HLH_glszm_GrayLevelNonUniformity, and wavelet-LLL_glcm_Idmn (Figure 8A and Table 3). There was no significant correlation between the radiomic features and the relative serum expression of *TP53* (Figure 8B). In summary, the combination of RT–qPCR and IHC results indicated that some of the radiomic features were correlated with *TP53* expression.

**Table 3:** Correlations between PCAT radiomic features and *TP53.*

Radiomics features	*R_s_*	*p*
First-order features		
wavelet-HLH_firstorder_Mean	0.624	0.003
wavelet-LHH_firstorder_Skewness	0.603	0.005
wavelet-LHL_firstorder_Energy	0.530	0.016
wavelet-LHL_firstorder_TotalEnergy	0.530	0.016
Textural features		
gradient_gldm_LargeDependenceHighGrayLevelEmphasis	0.617	0.004
original_glcm_ClusterProminence	0.507	0.022
original_glcm_ClusterTendency	0.504	0.023
original_gldm_DependenceEntropy	0.507	0.023
original_glrlm_RunEntropy	0.529	0.016
original_glszm_ZoneEntropy	0.561	0.010
wavelet-HHH_gldm_DependenceVariance	0.565	0.009
wavelet-HLH_glcm_ClusterShade	0.569	0.009
wavelet-HLH_glszm_GrayLevelNonUniformity	0.534	0.015
wavelet-LLL_glcm_Idmn	0.574	0.008

## 4 Discussion

In this study, we screened the candidate genes *HLA-DRB1*, *TP53*, and *CCR9* by bioinformatics to investigate their roles in the occurrence and development of CAD combined with T2DM. We found that high expression of *TP53* may play a role in the disease process of CAD combined with T2DM. Comprehensive analyses showing the association of the radiomic features of PCAT with the key gene *TP53* in patients with CAD combined with T2DM can more comprehensively and accurately assess the anatomical and functional changes in coronary arteries in patients with CAD combined with T2DM, which is valuable for accurately predicting the disease risk of CAD combined with T2DM and highlights the critical role of this gene, which may provide clues for the discovery of new imaging biomarkers. Although the study sample size was small and included only patients who underwent CABG, the complexity and uniqueness of the sample acquisition method warrant attention. This dataset offers unique scientific insights, particularly in revealing significant associations between PCAT radiomic features and *TP53* expression patterns, opening new perspectives for understanding the pathogenesis of CAD combined with T2DM. However, the findings should be regarded as preliminary evidence, and future validation requires larger, multicentre cohort studies to assess their generalizability.

The mechanism of CAD combined with T2DM is complex and involves various biological processes, including the inflammatory response, metabolic disorders, and apoptosis, amongst which the inflammatory response plays a key role in the development and progression of CAD and T2DM (17), leading to the coronary artery stenosis of patients with CAD combined with T2DM being more severe (18). The pathological state of PCAT leads to the release of proinflammatory mediators, which in turn trigger the recruitment of inflammatory cells and promote the onset and progression of coronary atherosclerosis (19, 20), in which changes in the expression of key genes are thought to be important players. The results of our previous study revealed a significant correlation between PCAT radiomic features and gene expression in CAD patients, highlighting the potential of CCTA-based radiomic features as a noninvasive CAD risk assessment tool (21). With the development of radiomics, inflammatory changes in PCAT can be detected by CCTA-based radiomics. Currently, CCTA is used to quantify the risk of CAD by measuring the perivascular fat attenuation index and epicardial adipose tissue (EAT) density, thickness and volume. Inflammation in coronary arteries releases signals to inhibit PCAT production, and CCTA can observe the attenuation characteristics of PCAT, capture dynamic inflammation in coronary arteries, and track changes in coronary artery inflammation (14). Radiomic first-order features are able to delineate and quantify each voxel of the region of interest in detail, reflecting the details of PCAT changes at the microscopic level (22). Morphological features describe the structural morphology of the overall region of interest to assess the inflammatory response of the coronary arteries and their surrounding lipid alterations (23). Texture features help to capture more complex organisational information by analysing the 3D spatial structure from pixel to pixel in the image, revealing the structural logic hidden in the spatial image (24). By combining these three features, not only can the macroscopic pixel spatial distribution of PCAT be meticulously analysed but also the microscopic local details can be explored in depth, thus achieving a comprehensive assessment of the heterogeneity of PCAT. Dong et al. (25) reported that radiomic features of PCAT based on CCTA provide timely diagnostic tools for patients with CAD combined with T2DM in the clinic and help reduce the risk of death due to cardiovascular disease.

Gene-level validation of candidate genes in PCAT revealed statistically significant differences for *TP53* across both groups, whereas *HLA-DRB1* and *CCR9* showed no such differences. Previous research indicates that *CCR9* and *HLA-DRB1* may play crucial roles in CAD and T2DM progression by influencing inflammatory responses and insulin resistance, respectively (26–29). However, our findings indicate that these genes do not exhibit key expression characteristics in CAD combined with T2DM. The specific mechanisms require further investigation through expanded sample size studies to validate their roles in the pathogenesis of CAD combined with T2DM. As an important oncogene whose function involves cell cycle regulation, DNA repair, and apoptosis, *TP53* plays an critical role in various pathological conditions, such as coronary heart disease and inflammation (5). The inhibition of *TP53* expression can slow the process of coronary atherosclerosis (30). In this study, the unit cell area of PCAT adipocytes was significantly larger in the T2DM group than in the control group, and the expression level of *TP53* in PCAT adipose tissue was significantly greater than that in the control group, suggesting that the progression of lesions in the coronary arteries of patients with CAD combined with T2DM is more severe, the degree of coronary artery stenosis is more severe, and *TP53* is closely related to the occurrence of CAD combined with T2DM. Previous reports have shown that *TP53* expression is increased in the adipose tissue of T2DM model mice, that high expression of *TP53* may lead to an impaired insulin signalling pathway, and that increased inflammation leads to insulin resistance, which in turn exacerbates the pathological state of T2DM (31, 32). Therefore, it is hypothesized that inflammatory changes occur in the coronary PCAT of patients with CAD combined with T2DM, with a significant increase in the expression of *TP53* in the PCAT, which favours an increased inflammatory response and insulin resistance, leading to mitochondrial damage and apoptosis; moreover, a tight bidirectional interaction was found between the pathological state of the PCAT and the coronary wall because the PCAT is much closer to the coronary arteries (19). High expression of *TP53* by PCAT around coronary arteries in patients with CAD combined with T2DM counteracted the coronary vasculature, exacerbated the existing inflammatory response of the coronary vasculature, and thus further exacerbated the degree of coronary atherosclerosis in patients with CAD combined with T2DM. *TP53* may be a key gene in the disease process of CAD combined with T2DM, and its role in inflammation and metabolic dysfunction deserves further exploration.

This study observed higher CK levels in the CAD combined with T2DM group compared to the CAD group alone, suggesting that chronic hyperglycaemia may exert an additional effect independent of traditional risk factors along the “creatine kinase-myocardial injury” axis. Patients with T2DM exhibit chronic low-grade inflammation, oxidative stress, and microvascular dysfunction. These factors not only exacerbate myocardial ischemia but may also cause CK leakage from cardiac and skeletal muscle by increasing cell membrane permeability or directly damaging myocytes (33, 34). Future studies with larger sample sizes are necessary to determine whether elevated CK truly reflects myocardial injury.

PCAT exhibits major metabolic abnormalities in the pathological process of patients with CAD combined with T2DM, releasing a variety of bioactive mediators, including proinflammatory cytokines (*IL-6*), adipokines (leptin), and vasoactive substances, through a paracrine mechanism; these bioactive molecules locally build a unique pathological microenvironment, which directly affects the coronary arterial wall through the diffusion of vascular epithelial cell function (35). ELISAs of the candidate genes *HLA-DRB1*, *TP53*, and *CCR9* revealed that the expression levels were not significantly different between the two groups, suggesting that *HLA-DRB1*, *TP53*, and *CCR9* in the peripheral blood did not significantly change during the pathological process of CAD combined with T2DM and that serum gene testing alone could not clearly differentiate the gene expression between patients with CAD and those with CAD combined with T2DM. The role of *HLA-DRB1*, *TP53*, and *CCR9* in predicting the risk of CAD combined with T2DM needs to be analysed comprehensively at different levels. The serum protein levels of *HLA-DRB1* and *CCR9* have been less well studied, and their pathological roles are more likely to involve local tissues or gene polymorphisms. Notably, genetic polymorphisms in *HLA-DRB1* are more strongly associated with T2DM or CAD than are changes in serum protein levels (36). *CCR9* has been less well studied in CAD or T2DM, and most studies have focused on immune cell surface receptor function rather than its soluble form in serum (37). Some studies have shown that serum levels of p53 antibodies may be elevated in patients with T2DM, but this result has not been widely validated in patients with CAD combined with T2DM, and the serological results of *TP53* are controversial in metabolic diseases and may vary depending on the stage of the disease or the method of detection (38).

In this study, the first-order and textural features of PCAT radiomics were closely related to the abnormal expression of *TP53*. In CAD patients, the inflammatory response leads to a decrease in adipocyte lipid accumulation and an increase in the aqueous phase of PCAT (14, 39), and ultimately, adipocytes and other inflammation-affected normal cell somatostatin were altered in the PCAT. Similarly, the combination of CAD with T2DM increases the release of proinflammatory mediators, thus leading to the alteration of first-order features of PCAT. This change leads to alterations in the first-order characteristics of PCAT. Patients with CAD may show structural alterations of PCAT, with increased local density asymmetry compared to unaffected adipose tissue, leading to spatial distribution inhomogeneity (40), which is exacerbated by the combination of T2DM, ultimately leading to alterations in the textural features of PCAT. First-order features and texture features can respond to changes in CAD combined with T2DM, and high expression of *TP53* can further exacerbate the degree of coronary atherosclerosis in patients with CAD combined with T2DM. These results suggest that the use of radiomic features can predict abnormalities in differential gene expression. With advances in radiomics, it is now possible to extract a large amount of quantitative information from imaging data that cannot be recognised by human readers, thereby identifying imaging patterns of significant clinical value. Radiomic features can predict both potential tumour phenotypes independently associated with tumour biology and clinical prognosis (41) and be used for risk prediction for adipose tissue fibrosis and microvascular remodelling (14). Linking features of these imaging data to the expression status of potential tissue biology gene markers could result in a more precise assessment of disease activity in patients and may provide new insights into disease mechanisms.

## 5 Limitations

Currently, this study faces several challenges and limitations. First, as a single-centre study with a small sample size, its findings serve only as preliminary evidence and may not fully represent broader populations. Further multicentre studies and in-depth development projects are needed to confirm the clinical utility of these imaging biomarkers. Second, this observational study only demonstrates an association between radiomic features and *TP53* expression. Future mechanistic studies, including *in vivo* and *in vitro* experiments, are needed to elucidate the causal relationships and potential biological pathways linking *TP53*, inflammation, insulin resistance, and PCAT radiomic features. Finally, whilst the key gene *TP53* and radiomic features proposed in this study show promise, rigorous external validation is required to obtain generalizable results before translating them into clinical early warning models.

## 6 Conclusion

In summary, the results of the present study demonstrated that *TP53* expression was significantly elevated in PCAT of patients with CAD combined with T2DM, a finding that highlights the role of *TP53* in the disease progression of CAD combined with T2DM. The radiomic features of PCAT in patients with CAD combined with T2DM are closely correlated with aberrant expression of *TP53*, suggesting the possibility of utilising radiomic features as predictive indicators of gene expression and using imaging biomarkers as predictive biomarkers for abnormal gene expression. This study exploits the association between radiomic features and gene expression by integrating them, which in turn offers the possibility of noninvasive prediction of disease onset in CAD combined with T2DM. We also provide a new perspective for the development of noninvasive predictive models for a more comprehensive understanding of the disease state and more accurate new imaging biomarkers for early diagnosis and assessment of response to therapy, as well as potentially contributing to the early identification of high-risk patients with CAD combined with T2DM in clinical practice.

## Data Availability

The datasets presented in this study can be found in online repositories. The names of the repository/repositories and accession number(s) can be found in the article/supplementary material.

## Footnotes

^1^ https://www.ncbi.nlm.nih.gov/gds

^2^ http://sangerbox.com/home.html

^3^ https://david.ncifcrf.gov/
